# Feeding *Bacillus-*based probiotics to gestating and lactating sows is an efficient method for improving immunity, gut functional status and biofilm formation by probiotic bacteria in piglets at weaning

**DOI:** 10.1016/j.aninu.2023.03.003

**Published:** 2023-03-29

**Authors:** Paweł Konieczka, Karolina Ferenc, Jens N. Jørgensen, Lea H.B. Hansen, Romuald Zabielski, Jarosław Olszewski, Zdzisław Gajewski, Magdalena Mazur-Kuśnirek, Dominika Szkopek, Natalia Szyryńska, Krzysztof Lipiński

**Affiliations:** a Department of Poultry Science, University of Warmia and Mazury in Olsztyn, Olsztyn, Poland; b Department of Animal Nutrition, The Kielanowski Institute of Animal Physiology and Nutrition Polish Academy of Sciences, Jabłonna, Poland; c Center for Translational Medicine, Warsaw University of Life Sciences, Warszawa, Poland; d Chr. Hansen A/S, Animal Health and Nutrition, Hoersholm, Denmark; e Department of Animal Nutrition and Feed Science, University of Warmia and Mazury in Olsztyn, Olsztyn, Poland; f Department of Histology and Embryology, Faculty of Veterinary Medicine, University of Warmia and Mazury in Olsztyn, Olsztyn, Poland

**Keywords:** Probiotic, Gut function, Immune status, Biofilm, Piglet, Sow

## Abstract

The effects of dietary probiotic supplementation with viable *Bacillus subtilis* and *Bacillus amyloliquefaciens* spores on sow performance, immunity, gut functional status and biofilm formation by probiotic bacteria in piglets at weaning were investigated. Ninety-six sows reared in a continuous farrowing system for one full cycle were fed gestation diets during the first 90 d of pregnancy and lactation diets until the end of lactation. The sows were fed a basal diet without probiotics (control; *n* = 48) or a diet supplemented with viable spores (1.1 × 10^9^ CFU/kg of feed) (probiotic; *n* = 48). At 7 d of age, sucking piglets (*n* = 12/group) were provided prestarter creep feed until weaning at 28 d of age. The piglets in the probiotic group were supplemented with the same probiotic and dosage as their dams. Blood and colostrum collected from sows and ileal tissues collected from piglets on the day of weaning were used for analyses. Probiotics increased the weight of piglets (*P* = 0.077), improved the weaning weight (*P* = 0.039) and increased both the total creep feed consumption (*P* = 0.027) and litter gain (*P* = 0.011). Probiotics also improved the faecal score in the second (*P* = 0.013) week of life. The immunoglobulin G (IgG) concentrations in sow blood at farrowing and the IgM concentrations in piglet blood at weaning were higher in the probiotic group than in the control group (*P* = 0.046). The piglets from the probiotic-treated sows showed a higher IgM concentration in the ileal mucosa (*P* = 0.050) and a lower IgG concentration in the ileal mucosa (*P* = 0.021) compared with the piglets from control sows. The probiotic-treated piglets had a thicker ileal mucosa (*P* = 0.012) due to the presence of longer villi and larger Peyer's patches (*P* < 0.001). *B. subtilis* and *B. amyloliquefaciens* were detected in the probiotic-treated piglets but not the control piglets; these bacteria were present in the digesta and villus structures and formed structures resembling biofilms. Overall, *Bacillus*-based probiotic supplementation improves the health indices of sows and their piglets.

## Graphical abstract

**Figure:**
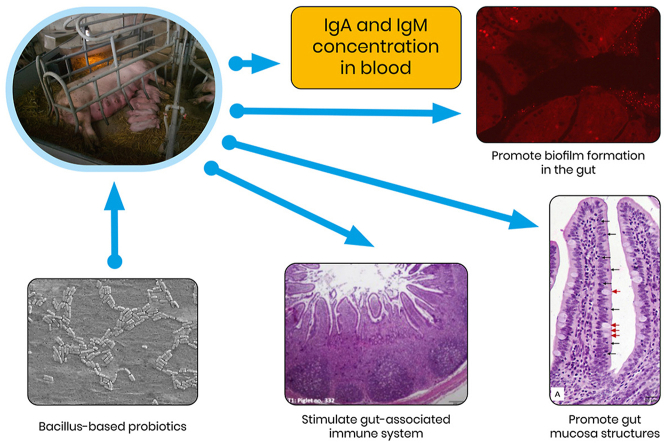

## 1 Introduction

The use of antibiotic growth promoters in animal feed was banned in the European Union in 2006 (Regulation (EC) No 1831/2003), and this ban has spurred interest in effective alternatives supporting the functional status of the gut in livestock (Barba-Vidal et al., 2019; Pandey et al., 2019). The group of alternative feed additives includes probiotics, prebiotics, acidifiers, medium-chain triglycerides, essential oils, and herbal extracts (Liao and Nyachoti, 2017; Omonijo et al., 2018; Upadhaya et al., 2018; van der Aar et al., 2017). Probiotic products appear to be suitable alternatives due to their potential to substantially improve the health status of pigs, and in contrast to antibiotics, probiotics produce bacteriocins, which are less associated with the potential development of resistance mechanisms in gut-resident bacteria (Quach et al., 2021). Probiotics are defined as live microorganisms that confer health benefits to the host by affecting bacteria in the gastrointestinal tract (Parker, 1974). The mechanisms of action of probiotics include the production of antimicrobial compounds, including vitamins, stabilizing microbial communities, restoring their natural balance, and the modulation of enzymatic activity in the gastrointestinal tract, which indirectly affects the growth and development of animals (Al-Shawi et al., 2020; Liu et al., 2018). A beneficial effect of probiotic supplementation on the growth performance of pigs has been reported by many researchers (Baker et al., 2013; Giang et al., 2011; Huang et al., 2004; Jaworski et al., 2017; Yu et al., 2008). Beneficial gut bacteria increase resistance to intestinal infections caused by pathogens such as *Escherichia coli*, *Salmonella*, *Campylobacter* and *Clostridium* (Alloui et al., 2013; Jankowska et al., 2008). Numerous strains of probiotic bacteria stimulate the activity of gut-associated lymphoid tissue (GALT) by increasing the number of T cells in the intestinal mucosa and immunoglobulins (IgA) secretion, which results in improvements in disease resistance in animals (Clancy, 2003). Probiotic feed additives stimulate antibody production and consequently boost the immune system naturally (Barba-Vidal et al., 2019; Dong et al., 2013; Naqid et al., 2015). The mechanisms of innate immunity are often weakened in gestating sows, and dietary supplementation with probiotics has been shown to contribute to reducing the incidence of disease (Jang et al., 2013; Kritas et al., 2015; Laskowska et al., 2019) and thus improves the health status of animals, colostrum quality and piglet performance (Alexopoulos et al., 2004; Kritas et al., 2015; Stamati et al., 2006). The use of probiotic-based products in swine has been widely investigated (Lambo et al., 2021); however, the currently available conclusive data are scarce because the gut environment, a potential target of probiotic action, is characterized by complex interactions between the gut microbiome and the host, including competition for nutrients and for adhesive receptors in the gut, and activities associated with the stimulation of host immunity (van Dijk et al., 2018). Faecal and/or digesta microbiota proliferation has been widely shown to be modified by probiotic supplementation in swine (Ding et al., 2020). In fact, the environmental complexity and continuous flow within the gastrointestinal tract are responsible for the low probiotic bioavailability to the host and limited intestinal colonization, particularly the formation of biofilms on the gut surface, which strongly determines their actions on the host (Wang et al., 2020b). Therefore, a more in-depth investigation is needed to understand the specific mechanism for enhancing the successful application of probiotics in swine nutrition.

The research hypothesis postulates that the addition of a probiotic containing viable spores of *Bacillus subtilis* and *Bacillus amyloliquefaciens* to diets fed to gestating and lactating sows and to creep feed will improve the performance of sows and modulate the functions of the developing gut of their offspring. The aim of this study was to evaluate the immunity, gut functional status, and biofilm formation by probiotic bacteria in piglets at weaning and the performance of gestating sows fed probiotic-supplemented diets.

## 2 Materials and methods

### 2.1 Animal ethics statement

This experiment was approved by the II Local Ethics Committee for Animal Experimentation in Warsaw, Poland (nr WAW2/011/2021).

### 2.2 Animals and management

This experiment was performed with 96 sows (primiparous and multiparous) of the DanBred genetic line in a pig farm located in northeastern Poland (Ławki n/Ryn, Poland). The average parity of sows was 3.9. The animals were reared in a continuous farrowing system for one full cycle and divided into 2 groups (control and experimental) of 48 pigs each. Pregnant sows were housed in group pens. During lactation, the pigs remained in individual stalls. All animals remained clinically healthy during the study.

The sows were fed mashed diets, including gestation diets administered during the first 90 d of pregnancy and lactation diets administered from 90 d of pregnancy until the end of lactation (Table 1). The pregnant sows were fed a restricted feed ration. During lactation, all sows and piglets were provided the corresponding diet ad libitum. The sows in the untreated control group (control group) did not receive probiotics in the feed, and the diets of the treated experimental sows (probiotic group) were supplemented with probiotics at a dose of 400 g/t of diets (supplemented as an extra ration of the diets). The probiotic mixture contained 2.75 × 10^9^ CFU/g viable spores of *B. subtilis*–541 and *B. amyloliquefaciens–*516. Sucking piglets were provided prestarter creep feed starting from 7 d of age until weaning at 28 d of age (Table 2). The piglets in the control group were provided standard creep feed, whereas those in the probiotic group were fed a prestarter supplemented with the same probiotic as their dams. The following zootechnical parameters of the sows were investigated: body weight, feed intake, body condition score (BCS), backfat thickness and faecal score at the beginning of gestation, at 90 d of pregnancy, at farrowing and at the end of lactation. During gestation, the number and frequency of abortions were controlled. The litter performance traits analysed in this study were the number and birth weight of piglets born alive and weaned, feed intake, mortality, and faecal score. The body condition of the sows was assessed both visually and by recording back fat measurements. The body condition of the sow was assessed visually on a numerical rating of 1 to 4 points. A score of 1 point was used for very thin sows, a score of 4 points was given to fat sows, and the optimal score was assigned a value of 3 points. The sow backfat thickness at the 10th rib, 7.5 cm from one side of the backbone, was measured at d 90 of gestation, after farrowing (d 1 of lactation), and at weaning (d 28 of lactation) using a digital backfat indicator (Renco Corp., Minneapolis, MN, USA).

**Table 1:** Composition and nutritional value of sow diets (%, as-fed basis).

Item	Content	
	Pregnant sows	Lactating sows
Composition		
Barley	20.5	23.5
Triticale	12.5	10.0
Wheat	–	10.0
Oat	10.0	5.0
Corn	10.0	13.0
Wheat bran	20.0	3.5
Soybean meal	6.5	20.0
Sugar beet pulp	10.0	2.0
Apple meal (byproduct from juice production)	4.0	5.0
Soybean oil	0.5	2.0
Premix for pregnant sows 1	6.0	–
Premix for lactating sows 2	–	6.0
Nutritive value		
Metabolizable energy, MJ/kg	12.50	13.50
Crude protein	14.0	17.0
Lysine	0.67	1.03
Methionine + Cysteine	0.51	0.64
Threonine	0.53	0.68
Tryptophan	0.17	0.21
Calcium	0.95	0.85
Digestible phosphorus	0.48	0.42
Sodium	0.22	0.22

1 Composition per kilogram of the pregnant sow premix: 12.5% Ca, 3.6% P, 3.2% Na, 1.2% Lys, 0.3% Met, 1% Thr, 1,800,000 IU of vitamin A, 33,340 IU of vitamin D_3_, 2,000 mg of vitamin E, 27 mg of vitamin K_3_, 27 mg of vitamin B_1_, 72 mg of vitamin B_2_, 54 mg of vitamin B_6_, 0.45 mg of vitamin B_12_, 54 mg of folic acid, 180 mg of pantothenic acid, 360 mg of niacin, 8 mg of biotin, 7,000 mg of choline chloride, 1,280 mg of Mn, 2,400 mg of Zn, 2,400 mg of Fe, 400 mg of Cu, 32 mg of I, 8 mg of Se, an antioxidant, and xylanase + β-glucanase + phytase.

2 Composition per kilogram of the lactating sow premix: 13.3% Ca, 4.5% P, 3.3% Na, 4% Lys, 1.1% Met, 1.6% Thr, 1,800,000 IU of vitamin A, 33,340 IU of vitamin D_3_, 2,000 mg of vitamin E, 27 mg of vitamin K_3_, 27 mg of vitamin B_1_, 72 mg of vitamin B_2_, 54 mg of vitamin B_6_, 0.45 mg of vitamin B_12_, 54 mg of folic acid, 180 mg of pantothenic acid, 360 mg of niacin, 8 mg of biotin, 7,000 mg of choline chloride, 1,280 mg of Mn, 2,400 mg of Zn, 2,400 mg of Fe, 400 mg of Cu, 32 mg of I, 8 mg of Se, an antioxidant, and xylanase + β-glucanase + phytase.

**Table 2:** Composition and nutritive value of creep diet (%, as-fed basis).

Item	Content
Composition	
Barley	20.0
Wheat	20.0
Corn	10.0
Concentrate^1^	50.0
Nutritive value	
Metabolizable energy, MJ/kg	14.0
Crude protein	19.0
Lysine	1.62
Methionine + Cysteine	0.99
Threonine	1.04
Tryptophan	0.31
Calcium	0.73
Digestible phosphorus	0.45
Sodium	0.29

1 Composition per kilogram of concentrate: extruded full fat soybean, soybean concentrate, soybean flour, blood porcine plasma, blood porcine cells, potato protein, fish meal, whey, 12% lactose, 1.4% Ca, 0.85% P, 0.5% Na, 2.9% Lys, 1.25% Met + Cys, 1.75% Thr, 0.5 of Trp, 32,000 IU of vitamin A, 4,000 IU of vitamin D_3_, 330 mg of vitamin E, 6 mg of vitamin K_3_, 12 mg of vitamin B_1_, 16 mg of vitamin B_2_, 16 mg of vitamin B_6_, 0.1 mg of vitamin B_12_, 8 mg of folic acid, 120 mg of pantothenic acid, 80 mg of niacin, 0.4 mg of biotin, 1,400 mg of choline chloride, 80 mg of Mn, 280 mg of Zn, 160 mg of Fe, 280 mg of Cu, 2.4 mg of I, 0.6 mg of Se, an antioxidant, and xylanase + β-glucanase + phytase.

From weeks 1 to 4, the faecal scores were evaluated and recorded by the same individuals. The faecal score was determined by the average value of 10 sows from each group using a 4-point scoring system: 0 = firm, dry, small pellets; 1 = soft and shape; 2 = loose, unformed stool that assumes the shape of the container; and 3 = watery, liquid stool that can be poured. The faecal consistency of each litter (3 piglets from each of 12 sows per group) was measured daily from birth to weaning using a visual score ranging from 0 to 2 points (0 = no diarrhoea; 1 = diarrhoea; and 2 = severe diarrhoea).

### 2.3 Sample collection and laboratory analyses

The contents of dry matter (DM), crude ash, crude protein (CP), ether extract (EE), and crude fibre (CF) in feed samples were determined using standard methods (AOAC International, 2005).

#### 2.3.1 Colostrum and blood sampling and IgA, IgM, and IgG analyses

In the first farrowing cycle, 20 pregnant sows, 10 from the untreated control group and 10 from the probiotic group, were randomly selected for colostrum and blood sampling (the samples originated from the same sows) and were collected before the first suckling. Samples of sow colostrum and venous blood were obtained immediately after parturition (*n* = 10 in each group), and blood and ileal tissue samples were obtained from 3 female offspring piglets at weaning (i.e., on postnatal d 28; *n* = 30 in each group). Two 1-mL samples of colostrum were collected from each sow, filtered with cotton gauze, and deposited in Eppendorf tubes. The colostrum samples (total of 20 samples) were stored at –80 °C until further analysis. Before analysis, the thawed samples were centrifuged at 2,000 *× g* and 4 °C for 10 min, and the supernatant was collected for analysis. Five millilitres of venous blood were collected in tubes without anticoagulant (sow and piglet blood were collected from the subclavian vein; 80 samples total) and centrifuged at 2,000 *× g* for 10 min to obtain approximately 2.5 mL of blood plasma. Plasma aliquots were collected in 5 Eppendorf tubes and stored at –80 °C until the assay. The concentrations of immunoglobulins were measured using ELISA kits according to the manufacturer's instructions. The dilutions of colostrum and sow and piglet plasma were set experimentally (Table S1).

#### 2.3.2 Ileal tissue sampling for IgA, IgM and IgG and histological analyses

**Fig. 1:**
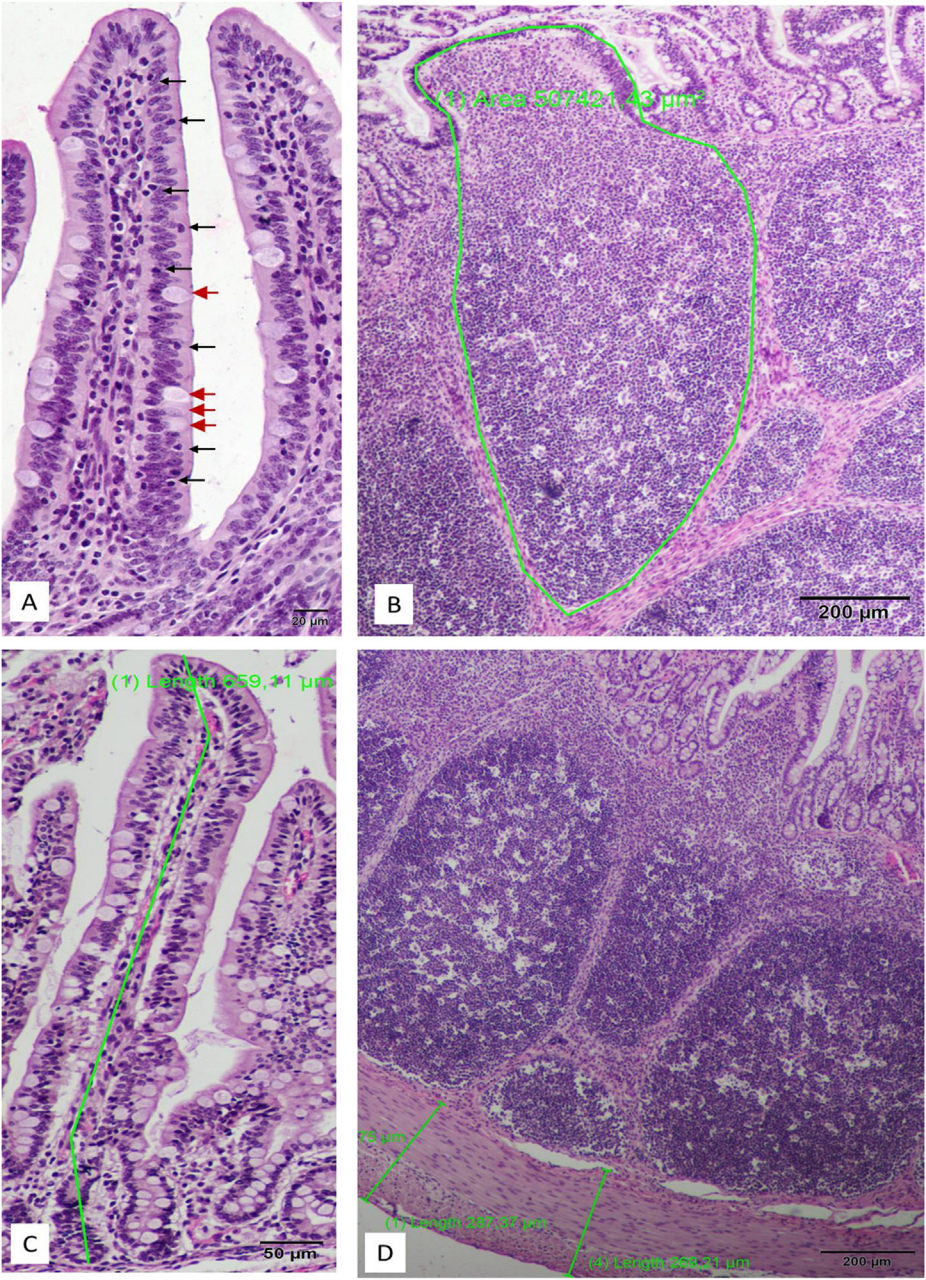
Control group images explaining the methodology of histological measurement in the ileum. (A) The goblet cells (GC; red arrows) and intraepithelial leukocytes (IEL; black arrows) in the epithelium were recognized by localization, shape, size, and cytoplasm pattern. (B) The average size of Peyer patches was measured only in the Peyer's patch sections showing full, uncut structures (green line shows the area of interest). (C) Mucosa thickness was expressed as a sum of villi length and crypt depth. (D) Measurement of muscularis layer thickness.

One female piglet of approximately middle weight from each litter in the control and probiotic groups (in each group *n* = 10) was sacrificed by barbiturate overdose to obtain ileal tissues for immunoglobulin ELISAs and histological analysis. Whole sections of the anterior ileum (length of 20 cm) were immediately flushed with PBS at room temperature and gently dried with a paper towel, and equal amounts of scraped mucosa were distributed into 6 Eppendorf tubes and frozen in liquid nitrogen. Afterwards, the scraped tissues were stored at –80 °C until analysis. After thawing, ileal mucosa samples were homogenized in an ice bath using a mechanical homogenizer. A total of 0.2 g of homogenate was diluted in 0.5 mL of PBS, and after 10 min of mixing, all the samples were centrifuged (20,000 *× g* and 4 °C for 5 min). The supernatant was collected for analysis. Immunoglobulin concentrations were measured using ELISA kits according to the manufacturer's instructions. The dilution of the samples (colostrum as well as sow and piglet plasma) for ELISA assays was set experimentally (Table S1). Whole sections of the ileum with a length of at least 5 cm were immediately flushed with PBS at room temperature, placed in 100-mL containers containing 4% buffered formalin and sealed tightly. After 48 to 72 h, the formalin was removed, and the samples were incubated with ethanol (70%). The samples used for histology were stored at room temperature until histology slide preparation and microscopy analysis. Whole tissue intestinal samples were fixed using a tissue processor (Leica TP1020, Kawa.ska, Zalesie Górne, Poland) (dehydration in increasing concentrations of ethanol, xylene washing, and paraffin embedding). Samples were cut into 5-μm sections (Leica RM2255 microtome, Kawa.ska, Poland) and processed using the standard haematoxylin and eosin staining protocol (Multistainer Leica ST5020, Kawa.ska, Poland). The thickness of the ileal mucosa, villus length, crypt depth, and areas of Peyer's patches in the ileum were measured at 4× magnification using an optical microscope (Olympus B×43) equipped with a digital camera and CellSens v.3 (Olympus, Tokyo, Japan) software (Olszewski et al., 2021). The size of microvilli was measured at 100× magnification. Only complete sections of Peyer's patches were selected for analysis (both the basal round-shaped part and the top part must have been intact and free of artefacts). The cell numbers in Peyer's patches were counted using MicroImage (Olympus) software. The percentage of goblet cells in the epithelium and the percentage of intraepithelial leukocytes (IEL) were measured using the method described below. First, the epithelial lineage area was discriminated on the villus using the area of interest option (MicroImage, Metro Manila, Philippines). Second, the total number of cell nuclei was measured in the marked area of interest (MicroImage) (Fig. 1). Third, the goblet cells and IEL were counted manually in the area of interest. This procedure was repeated for 6 to 10 villi in sections on one slide, and the percentages of goblet cells and intraepithelial leukocytes relative to all epithelial cells were then calculated. For each slide, the villi were selected for cell counting based on their structural integrity, profile shape, size, and morphological consistency (Fig. 1). For each intestinal segment, at least 5 slides were analysed (Olszewski et al., 2021).

#### 2.3.3 Ileal tissue sampling for fluorescence in situ hybridization (Histo-FISH)

The presence of *B. subtilis* and *B. amyloliquefaciens*, which are part of the supplemented probiotic mixture, and their spatial organization in the ileum were analysed using the Histo-FISH protocol. All steps of the procedure, including tissue fixation, embedding, sectioning, hybridization, and FISH, were performed according to the Histo-FISH protocol described by Madar et al. (2015) and the modification described by Konieczka et al. (2018). Ileal samples were collected from 24 piglets (*n* = 12 piglets per group), that is, one middle-weight female piglet per litter. Briefly, a whole section of ileum with a length of at least 5 cm was gently excised (with the internal content). Thereafter, the samples were immediately fixed with freshly prepared Carnoy's solution (60% ethanol, 30% chloroform and 10% glacial acetic acid at volume) for 4 h at 4 °C. A probe for *B. subtilis* and *B. amyloliquefaciens* (5ʹTCGCTTCCTGTACTGAATCTTCCATGT3ʹ), which was complementary to the bacteria in the probiotic mixture added to the diet, was used for hybridization. The sequence of the probe was designed by Amplicon sp. z. o. o. (Wrocław, Poland) and was synthesized and delivered by Sigma–Aldrich (Sigma Chemical Co., St. Louis, MO, USA). The probe was tagged at the 5ʹ-end with 101 acid chloride fluorochrome dye (Texas red; Txrd), with excitation and emission wavelengths of 587 and 647 to 670 nm (red), respectively. The 4ʹ,6-diamidino-2-phenylindole dye, with an excitation wavelength of 365 nm and an emission wavelength of 445 to 450 nm, was used as a counterstain. Tissue sections from each piglet were analysed in duplicate. The sections were examined using a Zeiss AxioImager fluorescence microscope equipped with a digital camera (Carl Zeiss, Germany).

### 2.4 Statistical analysis

The data were analysed to ensure a normal distribution before statistical analyses were performed. For statistical comparisons, the percentages of IEL and goblet cells were transformed by the arcsine of the square root. The mean values, SD and SEM in the tables are the original values.

The homogeneity of variance in 2 groups was verified using Levene's test, and when the data did not display a normal distribution or homogeneity of variance, they were subjected to a Box–Cox transformation before statistical comparisons. This transformation was applied to the following indices.

1. IgA concentration in the sow colostrum: *Y*’ = (*Y*^−0.440027^ − 1)/−0.440027,
2. IgA concentration in the sow blood plasma: *Y*’ = (*Y*^−0.489506^ − 1)/−0.489506,
3. IgM concentration in the sow blood plasma: *Y*’ = (*Y*^0.887631^ − 1)/0.887631,
4. IgA concentration in the piglet blood plasma: *Y*’ = (*Y*^0.179345^ **−** 1)/0.179345,
5. IgM concentration in the piglet blood plasma: *Y*’ = (*Y*^−0.233702^ − 1)/−0.233702,
6. IgG concentration in the piglet blood plasma: *Y*’ = (*Y*^0.290195^ − 1)/0.290195.

*T* tests for independent samples were performed using STATISTICA v13 software (StatSoft, Krakow, Poland). The Mann–Whitney U test was used for comparisons of muscle layer thickness parameters between groups. *P* ≤ 0.05 was considered to indicate a significant difference, and 0.05 < *P* ≤ 0.10 was considered to indicate near significant trends. The results are presented in the Tables and Figures as mean values with pooled SEM.

## 3 Results

### 3.1 Animal performance

The sows fed diets supplemented with *Bacillus*-based probiotics exhibited reductions in back fat loss (*P* ≤ 0.05) and body weight loss during lactation (*P* < 0.001) compared with the animals from the control group (Table 3). No differences in the average daily feed intake of sows, farrowing rate, weaning-oestrus interval or non-productive days were observed between groups.

**Table 3:** Sow performance.

Item	Control^1^	Probiotic^2^	SEM	*P-*value^3^
Number of sows	96	96		
Average parity	3.9	4.0		
Body weight, kg				
Gestation	241.3	239.4	4.986	0.847
Farrowing	305.0	302.3	4.637	0.780
End of lactation	269.4	277.9	4.528	0.350
Loss during lactation	35.59^B^	24.47^A^	1.166	<0.001
Sow back fat, mm				
Gestation	15.88	15.63	0.368	0.736
Farrowing	20.30	20.36	0.363	0.951
End of lactation	16.61	17.30	0.354	0.338
Loss during lactation	3.68^b^	3.05^a^	0.155	0.040
Sow body condition score				
Gestation	2.79	2.73	0.078	0.691
Farrowing	3.23	3.32	0.053	0.394
End of lactation unit	2.57^x^	2.77^y^	0.055	0.064
Change during lactation	0.66	0.55	0.052	0.282
Average daily feed intake, kg				
Gestation	3.18	3.16	0.012	0.601
Lactation	6.55	6.71	0.056	0.151
Farrowing rate, %	88.67	90.48	3.320	0.784
Weaning to the first oestrus interval, d	5.11	4.81	0.134	0.258
Non-productive days	12.48	8.81	1.674	0.276

1 No probiotic treatment.

2 The 2-strain probiotic supplement contained 2.75 × 10^9^ CFU/g viable spores of *B. subtilis*–541 and *B. amyloliquefaciens*–516.

3 Different superscripts in the same row indicate significant differences or trends (^AB^, *P* ≤ 0.01; ^ab^, *P* ≤ 0.05; ^xy^, 0.05 < *P* ≤ 0.10). The means were analysed using Tukey's test.

Probiotic administration tended to increase the average individual weight of live piglets (*P* = 0.077). Moreover, probiotics increased the weaning weight, total creep feed consumption, and piglet and litter weight gain (*P* ≤ 0.05). The probiotic preparation had no effect on the litter size or preweaning mortality (Table 4).

**Table 4:** Litter performance.

Item	Control^1^	Probiotic^2^	SEM	*P-*value^3^
Litter size, number per litter				
Total born	18.84	19.00	0.365	0.829
Alive born	17.18	17.43	0.333	0.710
Stillborn	1.34	1.23	0.136	0.679
Mummies	0.32	0.34	0.074	0.878
Intrauterine growth restricted piglets	2.41	2.16	0.203	0.542
After cross-fostering	14	14	–	–
Alive at d 7	12.82	12.89	0.052	0.519
Weaned	12.71	12.82	0.063	0.367
Preweaning mortality, %	9.25	8.44	0.447	0.367
Piglet age at weaning, d	25.59	25.77	0.137	0.509
Piglet body weight, kg				
Average individual weight of live-born piglets	1.27^x^	1.34^y^	0.019	0.077
Average weight of piglets after cross-fostering	1.54	1.50	0.023	0.410
Average individual piglet weight at weaning	6.60^a^	6.94^b^	0.081	0.039
Litter weight, kg				
At birth	21.72	22.97	0.403	0.123
At cross-fostering	21.57	21.03	0.326	0.410
At weaning	83.99^a^	88.93^b^	1.166	0.034
Litter body weight gain				
Total litter weight gain (cross-fostering till wean), kg	62.42^a^	67.90^b^	1.081	0.011
Average daily weight gain of piglets (cross-fostering till wean), g	198.2^a^	211.2^b^	2.957	0.027
Total creep feed consumption, kg	5.12^a^	5.42^b^	0.067	0.027

1 No probiotic treatment.

2 The 2-strain probiotic supplement contained 2.75 × 10^9^ CFU/g viable spores of *B. subtilis*–541 and *B. amyloliquefaciens*–516.

3 Different superscripts in the same row indicate significant differences or trends (^AB^, *P* ≤ 0.01; ^ab^, *P* ≤ 0.05; ^xy^, 0.05 < *P* ≤ 0.10). The means were analysed using Tukey's test.

No significant differences in the faecal scores of sows were observed between groups. The piglets in the probiotic group exhibited improved faecal scores in the second week of life (*P* ≤ 0.05) and tended to have increased faecal scores in the first (*P* = 0.059) and third (*P* = 0.09) weeks of life (Table 5).

**Table 5:** Faecal score^1^.

Item	Control^2^	Probiotic^3^	SEM	*P-*value^4^
Sow faecal score				
Week 1	0.07	0.18	0.035	0.110
Week 2	0.18	0.14	0.039	0.565
Week 3	0.23	0.18	0.043	0.602
Week 4	0.36	0.34	0.054	0.834
Litter faecal score				
Week 1	0.61^y^	0.34^x^	0.072	0.059
Week 2	0.50^b^	0.18^a^	0.064	0.013
Week 3	0.48^y^	0.25^x^	0.067	0.090
Week 4	0.41	0.34	0.054	0.534

1 Pen faecal scores (litter) range from 0 to 2 points: 0 (normal)–less than 10% of the pigs in the pen have loose or watery faeces, 1 (loose)–10% to 50% of the pigs in the pen have loose or watery faeces, and 2 (severe)–more than 50% of the pigs in the pen have loose or watery faeces. The sow faecal score ranged from 0 to 3 points: 0–firm stool, 1–soft and shape stool, 2–loose stool, and 3–watery stool.

2 No probiotic treatment.

3 The 2-strain probiotic supplement contained 2.75 × 10^9^ CFU/g viable spores of *B. subtilis*–541 and *B. amyloliquefaciens*–516.

4 Different superscripts in the same row indicate significant differences or trends (^ab^, *P* ≤ 0.05; ^xy^, 0.05 < *P* ≤ 0.10). The means were analysed using Tukey's test.

### 3.2 Concentrations of immunoglobulins in the colostrum, blood, and ileal tissue

No significant differences in the concentrations of immunoglobulins in sow colostrum and blood plasma were observed between the probiotic and control groups, with the exception of the IgG concentrations in blood plasma (Table 6). The IgG concentration in the sow blood plasma was higher in the probiotic group than in the control group (*P* = 0.046). The IgM concentration in the blood plasma of piglets at weaning was higher in the probiotic group than in the control group (*P* = 0.028) (Table 6). The concentrations of the other 2 immunoglobulins, IgA and IgG, did not show significant differences between different groups of piglets at weaning. Piglets from probiotic-treated sows showed higher IgM concentrations (*P* = 0.05) and lower IgG concentrations in the ileal mucosa homogenates than piglets from the control group (*P* = 0.021) (Table 6).

**Table 6:** Concentrations of immunoglobulins (IgA, IgM and IgG) in sow colostrum, sow and piglet blood plasma and piglet ileal tissue.

Item	Control^1^	Probiotic^2^	SEM	*P-*value^3^
Colostrum (sows)				
Number of sows (samples)	10	10		
IgA, mg/mL	28.41	27.81	3.60	0.921
IgM, mg/mL	9.49	10.12	0.78	0.700
IgG, mg/mL	132.7	120.2	12.0	0.616
Blood plasma at farrowing (sows)				
Number of sows (samples)	10	10		
IgA, mg/mL	3.43	4.38	0.94	0.234
IgM, mg/mL	5.96	6.56	0.64	0.660
IgG, mg/mL	18.08^a^	26.67^b^	2.20	0.046
Blood plasma at weaning (piglets)				
Number of litters	10	10		
Samples/litter	3	3		
IgA, mg/mL	0.439	0.510	0.037	0.248
IgM, mg/mL	0.688^a^	1.081^b^	0.078	0.028
IgG, mg/mL	8.82	10.16	0.64	0.212
Intestinal mucosal tissue at weaning (piglets)				
Number of litters	10	10		
Samples/litter	1	1		
IgA, μg/g tissue	412.7	301.8	47.5	0.254
IgM, μg/g tissue	5.08^a^	10.25^b^	1.34	0.050
IgG, μg/g tissue	45.82^b^	31.52^a^	3.22	0.021

1 No probiotic treatment.

2 The 2-strain probiotic supplement contained 2.75 × 10^9^ CFU/g viable spores of *B. subtilis*–541 and *B. amyloliquefaciens*–516.

3 Different superscripts in the same row indicate significant differences (^ab^, *P* ≤ 0.05). The means were analysed using Tukey's test.

### 3.3 Ileum structure in piglets at weaning

**Fig. 2:**
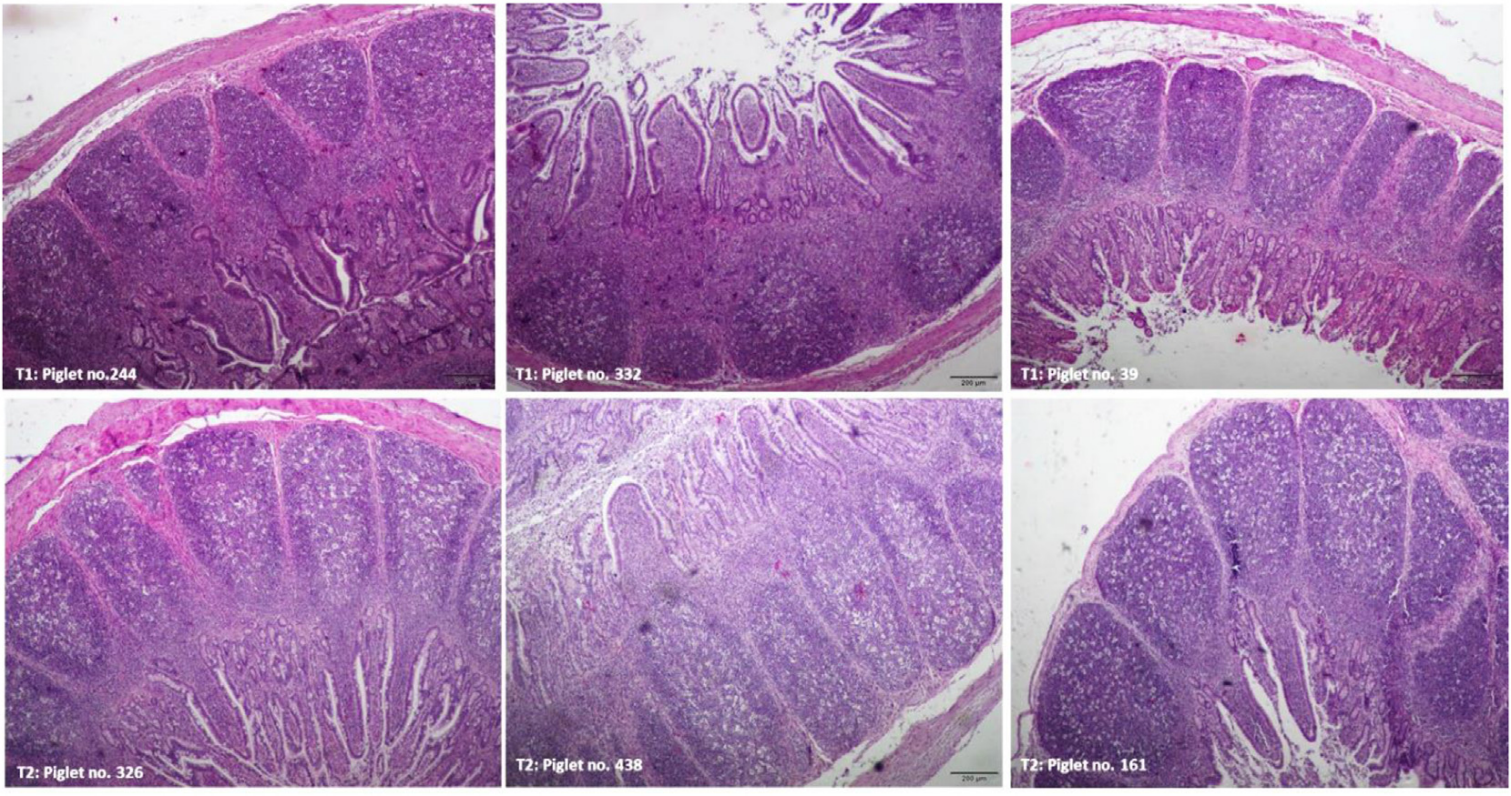
Representative images of the pig ileum showing the structure of the Peyer patches. The upper panel shows the images from piglets from the control group. The lower panel demonstrates the images from piglets from the probiotic-supplemented group. In the two panels, normal structure of the mucosa with a continuous epithelium layer is observed. Peyer patches in the probiotic group were larger in size (see Table 7 with numeric data), and the cells that built the Peyer patches seemed to be more differentiated than those in the control group. H&E staining, 4× magnification.

**Fig. 3:**
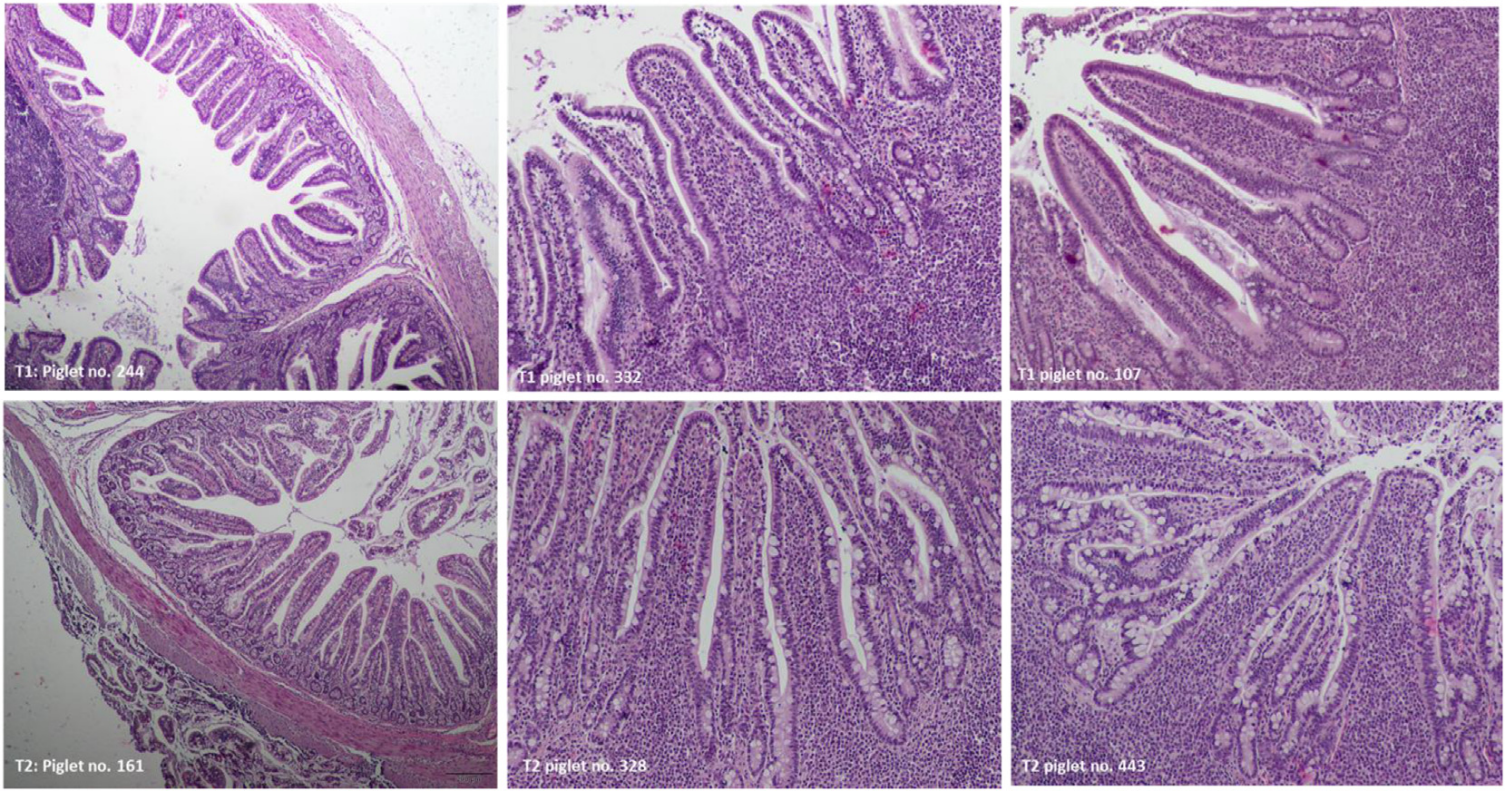
Representative images of ileal wall structure in piglets from the control group (upper panel) and the probiotic group (lower panel). In the two panels, normal structure of the ileal mucosa with continuous epithelium is observed. No foetal-type enterocytes were observed, and only adult-type enterocytes filled the epithelial lineage in the control and probiotic-supplemented groups, which suggests that in the 2 groups, the postnatal remodeling of the ileal mucosa was complete (Skrzypek et al., 2007). The ileal villi shown in the lower panel (probiotic-supplemented group) were longer and had more goblet cells (producing mucus) than the control group. H&E staining, 4× magnification (images on the left side), and 10× magnification (images in the middle and on the right side).

**Fig. 4:**
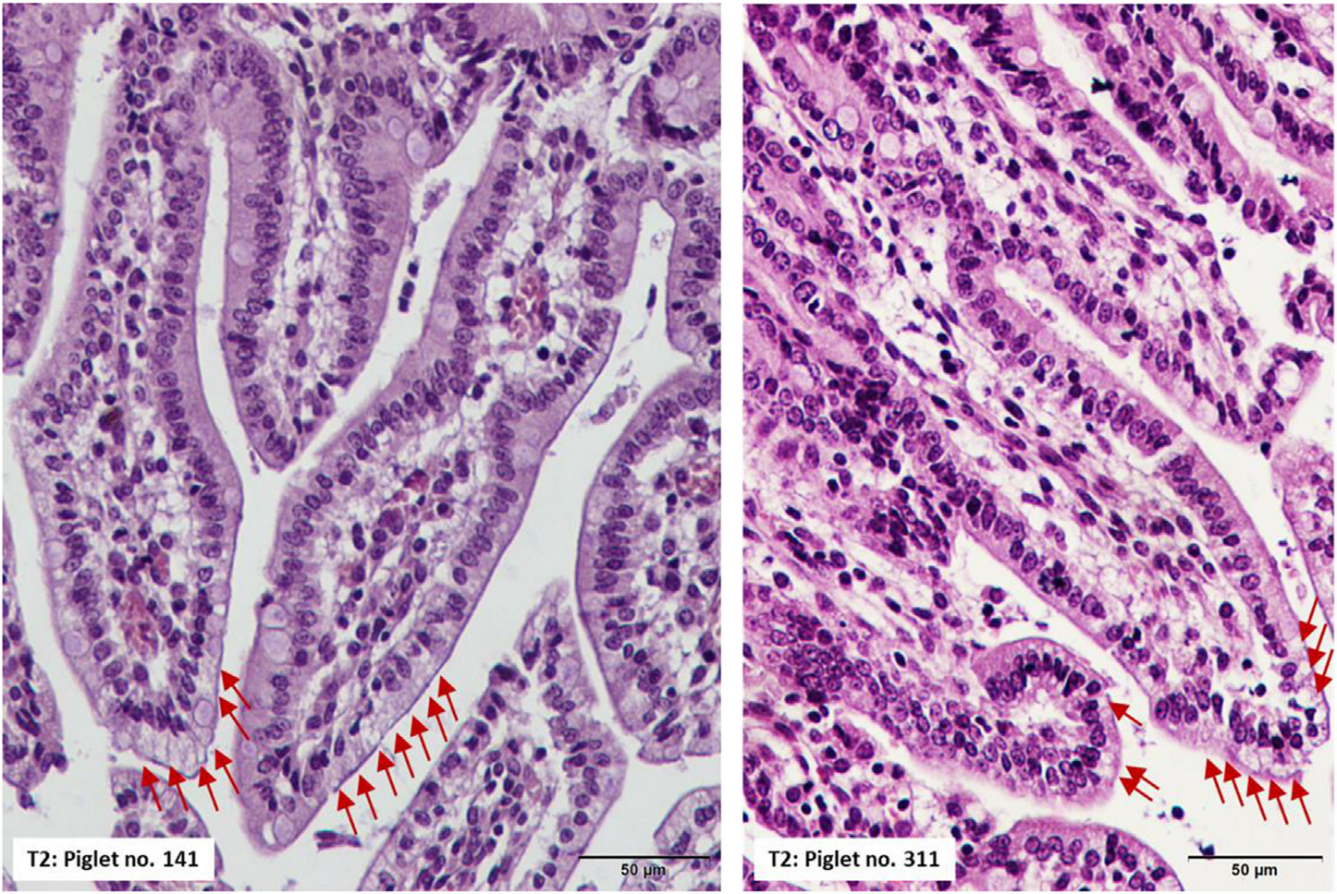
Foetal-type enterocytes (red arrows) were observed on the top of the ileal villi in two piglets from the probiotic group. No such enterocyte types were found in the control group (see Fig. 1A and C, and Fig. 3 upper panel).

A normal histological structure of the ileum was observed in piglets from the control and probiotic groups (Fig. 2, Fig. 3). No histological signs of inflammatory processes were observed, and the control and probiotic ileal mucosa groups had similarly low IEL counts, as shown in Table 7. The effect of supplementing the sow and piglet diets with probiotics on the ileal mucosa of piglets was observed; the mucosa was thicker due to the presence of longer villi (*P* = 0.012) because the crypt size was not different (Fig. 3, Table 7) and larger Peyer's patches (Fig. 2). The 19% difference in villus length was partially compensated by a 10% reduction in the microvillus size in the probiotic-treated group, although the continuity of the epithelium was well maintained in the 2 experimental groups (no morphological signs of enhanced cell apoptosis). The size of Peyer's patches was increased by 39% in the probiotic-supplemented group compared with the control group (*P* = 0.001) (Table 7). In the 2 piglets (sow no. 141 and 311) from the probiotic-supplemented group, we observed a few foetal-type enterocytes on the top of intestinal villi (Fig. 4). All other piglets from the control and probiotic-treated groups showed a well-developed ileal mucosal structure with no traces of foetal-type enterocytes in the gut epithelium (Fig. 3). However, the percentage of goblet cells was lower in the probiotic group than in the control group (*P* = 0.006) (Table 7).

**Table 7:** Histological analysis of the piglet ileal mucosa.

Item	Control^1^	Probiotic^2^	SEM	*P-*value^3^
Number of sows (samples)	10	10		
Sample/litter	1	1		
Microvillus length, μm	1.65^a^	1.50^b^	0.05	0.050
Villus length, μm	328.8^a^	390.1^b^	17.1	0.018
Crypt depth, μm	122.2	123.2	3.3	0.836
Villus to crypt ratio	2.70^x^	3.22^y^	0.171	0.055
Mucosal thickness, μm	443.1^a^	529.0^b^	17.9	0.012
Muscular layer thickness, μm	198.4	229.0	10.8	0.185
Average area of Peyer's patches, μm^2^	311.9^A^	434.4^B^	20.130	0.001
Percentage of intraepithelial leukocytes, %	16.15	15.04	0.93	0.663
Percentage of goblet cells, %	17.53^b^	12.34^a^	1.03	0.006

1 No probiotic treatment.

2 The 2-strain probiotic supplement contained 2.75 × 10^9^ CFU/g viable spores of *B. subtilis*–541 and *B. amyloliquefaciens*–516.

3 Different superscripts in the same row indicate significant differences (^AB^, *P* ≤ 0.01; ^ab^, *P* ≤ 0.05; ^xy^, 0.05 < *P* ≤ 0.10). The means were analysed using Tukey's test.

### 3.4 Visualization of the bacterial spatial organization in the gut

The visualization of the spatial organization of probiotic bacteria in the control piglets (referred to hereafter as 1 to 12) is shown in Fig. S1, whereas the probiotic bacterial structure in the probiotic-treated piglets (referred to hereafter as 13 to 24) is shown in Fig. S2. Overall, the bacteria *B. subtilis* and *B. amyloliquefaciens* were not readily detected in either the gut structures or the digesta of the control piglets. The only exceptions were piglets 3 and 11, in which these bacteria were visualized at low abundance and only in the digesta (Fig. S1). Cellular autofluorescence in the villus structures was also detected in piglets 8 and 9. The bacteria *B. subtilis* and *B. amyloliquefaciens* were detected in all of the probiotic-treated piglets (Fig. S2). These bacteria were present in the digesta and the villus structures (i.e., piglet 14). The bacilli mostly did not form structured biofilms, but in some cases (i.e., piglets 20, 21, 22 and 23), the bacteria closely covered villus edges, which were visualized as structures resembling biofilms. Probiotic bacteria were mainly present in aggregate-covered feed particles, which was particularly evident in piglet 24 (deep penetration of spaces between villi). Evidence of cellular autofluorescence was also observed.

## 4 Discussion

### 4.1 Animal performance

The intake of nutrients by a sow during different phases of the reproductive cycle affects the number of piglets born alive and the body weight of the piglets at birth and weaning (Whittemore, 2006). These parameters may be further affected by supplementation of the sow feed with biologically active components, including prebiotics and probiotics (Puzio et al., 2007; van der Aar et al., 2017; Zabielski et al., 2007). Probiotics are used for multiple purposes, such as decreasing the intestinal pH, inhibiting pathogenic microorganisms and modifying the host immune response. One of the mechanisms through which probiotics act is enhancing the intestinal barrier function, which leads to improvements in the health status and performance (Barba-Vidal et al., 2019; van der Aar et al., 2017). In the present study, the sows fed diets supplemented with the probiotic mixture were characterized by significant reductions in back fat and body weight loss during lactation and tended to display a better BCS at weaning. This result is consistent with those from previous studies conducted by Kritas et al. (2015). Alexopoulos et al. (2004) noted that dietary supplementation with a probiotic mixture containing *Bacillus licheniformis* and *B. subtilis* spores reduced the sow body weight loss during lactation. Similar results were also reported by Stamati et al. (2006). Other researchers found no differences in the body condition, weight loss during lactation or backfat thickness of sows receiving probiotics containing *Bacillus* during the reproductive cycle (Hu et al., 2021; Jeong et al., 2015; Taras et al., 2006). The differences between studies may be related to a large number of factors, including different probiotic bacterial strains, farm health status, and feed composition.

The early period of a piglet's life is a critical time when the gut and immune system have not fully developed. This immature development might contribute to decreased disease resistance in piglets and may seriously affect performance parameters (Konstantinov et al., 2008; Liu et al., 2014). In the present study, the individual body weight of piglets from probiotic-supplemented sows was higher at birth and weaning than that of piglets from control sows. The probiotic-supplemented piglets had a higher cumulative creep feed intake, an increased litter weight gain and a significantly improved scouring index. These effects, particularly the effects observed during the first and second postnatal weeks, may not be explained solely by the probiotic mixture administered to the piglets. Therefore, we hypothesized that the positive effects observed in our study during the first 2 postnatal weeks were due to sow supplementation, whereas the positive effects observed in subsequent weeks were due to the combination of maternal and piglet supplementation with probiotics. Analyses of the inclusion of the investigated probiotic strains in feed have recently shown that the *B. amyloliquefaciens* strain particularly enhances the ileal digestibility of amino acids in pigs (Blavi et al., 2019). Furthermore, *E. coli* challenge studies have shown that the *B. subtilis* strain improves the scouring index of piglets (Kim et al., 2019; Luise et al., 2019a). Baker et al. (2013) showed tendencies towards greater litter masses at birth and weaning for sows treated with probiotics. These positive effects have also been described by other researchers (Alexopoulos et al., 2004; Hayakawa et al., 2016; Jorgensen and Hansen, 2006). The probiotic supplementation of sows and their offspring led to a reduction in the incidence of liquid faeces (Taras et al., 2005; Zhang et al., 2020a). In contrast, Jeong et al. (2015) found that probiotic (*B. subtilis* and *Lactobacillus acidophilus*) supplementation had no effects on the litter body weight or piglet survival. Bohmer et al. (2006) and Wang et al. (2014) reported no differences in the weaning weight of piglets receiving probiotic preparations (*Enterococcus faecium* or *Lactobacillus johnsonii*). The efficacy of probiotic supplementation appears to be related to the type of bacterial strain used in the study or to the farm environment, which may mask the biological effects of probiotics on livestock.

### 4.2 Concentrations of immunoglobulins in colostrum, blood plasma and ileal mucosa scrapings

The immunoglobulin quantities in sow colostrum in the present study are consistent with previous reports (Markowska-Daniel and Pomorska-Mól, 2010). As reported previously by Curtis and Bourne (1971), IgG was the main immunoglobulin detected in colostrum and blood plasma of our sows; however, the colostral IgG concentration was several-fold higher than that in blood, 7.3-fold higher in control sows and 4.6-fold higher in probiotic-supplemented sows (although the differences were due to a significant increase in the blood IgG concentration in the probiotic-treated group). With the exception of the differences in the IgG level in blood plasma, significant differences in immunoglobulin concentrations were not observed between the sow samples.

The IgG, IgA, and IgM concentrations in piglet blood plasma showed similar patterns to those in sows (IgG > IgA = IgM); however, the concentrations in piglets were markedly lower than those in their dams (IgG: approximately 2-fold lower, IgA: approximately 8-fold lower, and IgM: approximately 7- to 8-fold lower). Similar concentrations of IgA and IgG in piglet blood plasma were reported by other researchers (Bourne, 1973; Curtis and Bourne, 1971; Perricone et al., 2020). In the piglet intestinal mucosa, the major immunoglobulin detected was IgA, but no differences in IgA concentrations were observed between the control and probiotic groups due to high intragroup variation. The mucosal IgG concentration was approximately 10-fold lower than the IgA concentration; however, the IgG concentration was significantly lower in the probiotic group than in the control group. The IgM concentration was lower than the concentrations of the other 2 immunoglobulins in the gut mucosa but was significantly higher in the probiotic group than in the control group. Notably, the plasma IgM concentration was also increased in the probiotic-supplemented piglets. We did not investigate direct cause-and-effect relationships but noted that long-term administration of the probiotic mixture modified the immunoglobulin profiles in the colostrum and blood (maternal and offspring samples) and resulted in parallel improvements in the sow and piglet performance.

### 4.3 Structure and immune barriers in the piglet ileum

The probiotic-supplemented piglets had a thicker ileal mucosa and larger Peyer's patcher than the piglets from the control sows, which suggested a direct trophic effect of probiotics on the gut mucosa and the mucosal immune system. This effect might be achieved first by coprophagy (piglets had free access to the faeces of probiotic-supplemented dams) and second by creep feed (the sow feeder was not accessible to piglets). The effect of colostrum/milk immunoglobulins was minimal, if any; however, we examined the pig intestines only at one time point, postnatal d 28. Therefore, these conclusions must be considered with caution.

The percentage of intraepithelial leukocytes (an indicator of subclinical inflammatory processes in the gut mucosa) was low in the 2 groups, confirming good biosecurity standards in the nursery unit and piglet health. Consistently, the percentage of goblet cells in the piglet ileum was relatively low in the 2 groups. At this age, a mean value of approximately 20% (of all epithelial cells set to 100%) was expected. Nevertheless, the percentage of goblet cells was lower in the probiotic-supplemented group than in the control group. The difference is unclear but may be related to the villus length and/or slower rebuilding of the gut epithelium. The villus length of the probiotic-treated piglets was increased by 19% compared with that of the control piglets; this effect might be attributed to increased stem cell proliferation in the crypt and/or reduced apoptosis. Cell proliferation in the crypts appeared to be unchanged, although the number of apoptotic cells along the villi was reduced (data not shown). This finding helps us at least partially understand the reduced percentage of goblet cells in the epithelium because the lifespan of goblet cells is markedly longer than that of enterocytes. This result may also help explain why some foetal-type enterocytes were still present at postnatal d 28 in 2 piglets. Slower rebuilding of the gut epithelium (Mickiewicz et al., 2012; Skrzypek et al., 2007, 2018) does not contradict the hypertrophic effect on gut immune structures (i.e., Peyer's patches), as reported recently (Olszewski et al., 2021). Probiotic bacilli appear to selectively stimulate the development of immune structures in the ileum. This suggestion is supported by significantly elevated concentrations of IgM (produced chiefly by Peyer's patch B lymphocytes) in the ileal mucosa of probiotic-treated piglets, which indicates enhanced development of Peyer's patches (Furukawa et al., 2020). We speculated that a shift in energy utilization within the gut mucosa might have occurred: more energy for the gut immune system and less energy for the gut epithelium. This hypothesis may explain why fewer goblet cells and some foetal-type enterocytes were still present on top of the villi in the 2 piglets in the probiotic-treated group. Probiotic bacteria interact with many cell populations, including epithelial cells, dendritic cells, macrophages, and intraepithelial lymphocytes (Kalita et al., 2020).

The histological analysis of Peyer's patch structures suggests better organization in the probiotic-supplemented group than in the control group. At birth, Peyer's patches are small and do not show a clear structure in 3 sections (Olszewski et al., 2021). The final architecture of the diffuse lymphoid tissue of the gut appears at 6 postnatal weeks (Furukawa et al., 2020). IgA is the main immunoglobulin produced by Peyer's patch B lymphocytes (Andersen et al., 1999), which was also observed in the present study. Further studies examining histochemical markers of Peyer's patch-specific cells might provide additional insights into this aspect.

### 4.4 Morphology of the ileum versus piglet performance

The observed changes in the ileal mucosa (villus length, IEL, goblet cell count, and Peyer's patch architecture) coincided with an improved litter performance at weaning (e.g., body weight and scouring index). The piglets in the probiotic group ate more creep feed and showed greater weight gains compared with those in the control group. The consumption of creep feed is known to enhance maturation of the gastrointestinal tract structure and function and stimulate the rebuilding of the small intestinal epithelium (e.g., replacement of foetal-type enterocytes with adult-type enterocytes, changes in the brush border enzyme profile and activity, and changes in membrane transporter proteins) and the overall development of intestinal function (motility, secretion, and absorption of nutrients). These changes result in more efficient digestion of weaning feed and in reduced postweaning scouring because the piglet's digestive system is already adapted to solid feed. As shown in the present study, the improved piglet performance in the probiotic group compared with the control group might be supported by significantly thicker mucosa (longer villi = larger absorptive area and better conditions to digest and absorb nutrients; Wang et al., 2020a) and more developed Peyer's patches (larger Peyer's patch size and higher mucosal IgM concentration result in enhanced protection potential of the gut immune system; Olszewski et al., 2021).

The period of weaning and postweaning is considered a critical phase in the pig lifespan because it is usually associated with digestive disorders and contributes to increased mortality events to a large extent (Luise et al., 2019a). In this regard, probiotic supplementation, including *Bacillus*-based probiotics, may play a key role in supporting the growth, health, immunity, intestinal functionality, and microbial profile of pigs under both optimal and stress conditions (Luise et al., 2019b). However, the effect of probiotics on the pig gut environment depends on extensive interactions with the gut microbiome, including competition for nutrients, competition for attachment sites, and the production of bacteriostatic and bactericidal substances. Different studies have shown that spore-forming *Bacillus*-based bacteria survive in the gut environment and affect the bacterial composition of the host (Gu et al., 2021; Sun et al., 2021). Although we did obtain evidence showing that supplemented probiotic strains of bacteria built a structured biofilm in the piglet gut in the present study, their abundance visualized by Histo-FISH clearly confirmed that these bacteria were present in the lower parts of the piglet gut, which indicated that they germinated and were metabolically active to colonize this environment, but these findings were not observed in piglets from the control group. A study examining a broiler chicken model appears to confirm that supplementation with spore-forming *Bacillus*-based probiotics may affect both the bacterial composition and activity in the host gut (Konieczka et al., 2018, 2022). Overall, our data are in line of other reports indicating that *Bacillus*-based probiotic supplementation improve health status of sows and their offspring health status (Zhang et al., 2020b; Crespo-Piazuelo et al., 2022).

## 5 Conclusions

In conclusion, the feeding of *Bacillus*-based probiotics to gestating and lactating sows exerted a beneficial effect on their performance, as manifested in reductions in the back fat loss and body weight loss during lactation. The feeding of probiotics also tended to increase the weight of piglets, weaning weight, total creep feed consumption, and piglet and litter weight gain. Probiotic supplementation promoted IgG production in the sow blood and increased the IgM levels in the blood and ileal mucosa of piglets at weaning. Probiotic supplementation did not compromise the morphological structures in the piglet gut and appeared to exert trophic effects on the ileal mucosal barriers of offspring, resulting in a healthier gut. Probiotic bacteria were able to reach lower parts of the piglet gut and were able to form structures resembling biofilms. Our data indicated that *Bacillus*-based probiotic supplementation selectively stimulated the immune system in the ileal mucosa of offspring, resulting in a more functional gut immune system for weaning challenges.

## Author contributions

**Paweł Konieczka**: Investigation, Formal analysis, Data curation, Writing – original draft. **Karolina Ferenc**: Investigation, Data curation. **Jens N. Jørgensen**: Conceptualization. **Lea H.B. Hansen**: Conceptualization. **Romuald Zabielski**: Conceptualization, Investigation, Formal analysis, Data curation. **Jarosław Olszewski**: Investigation, Formal analysis. **Zdzisław Gajewski**: Investigation, Formal analysis. **Magdalena Mazur-Kuśnirek**: Investigation, Formal analysis. **Dominika Szkopek**: Investigation. **Natalia Szyryńska**: Investigation. **Krzysztof Lipiński**: Conceptualization, Formal analysis, Resources, Funding acquisition, Writing – review & editing.

## Declaration of competing interest

We declare that we have no financial and personal relationships with other people or organizations that can inappropriately influence our work, and there is no professional or other personal interest of any nature or kind in any product, service and/or company that could be construed as influencing the content of this paper.
