# Supplementary material for: Feeding Bacillus-based probiotics to gestating and lactating sows is an efficient method for improving immunity, gut functional status and biofilm formation by probiotic bacteria in piglets at weaning
Source: Anim Nutr. 2023 Mar 29;13:361–72. doi: 10.1016/j.aninu.2023.03.003 (PMC10300407; doi:10.1016/j.aninu.2023.03.003)
Supplement: Multimedia component 1 [file mmc1.docx]

**Table S1**

The dilution of the samples for ELISA immunoglobulin assays was set experimentally according to the concentration of immunoglobulins in the samples and the sensitivity of the test.

| ELISA kit | Producer and kit references | Dilution of samples | | | |  |
| --- | --- | --- | --- | --- | --- | --- |
|  |  | Colostrum | Sow plasma | Piglet plasma | Piglet ileal mucosa homogenate | |
| Pig IgA | MyBiosource Inc., USA, cat. no. MBS564138 (lot. 10) | 1:120,000 | 1:30,000 | 1:20,000 | 1:1,000 |  |
| Porcine IgM | MyBiosource Inc., USA, cat. no. MBS2512434 (lot. 9PZ8YEZRAP) | 1:120,000 | 1:30,000 | 1:20,000 | 1:100 |  |
| Porcine IgG | MyBiosource Inc., USA, cat. no. MBS2510566 (lot. D8SIB3XG1A) | 1:1,200,000 | 1:3,000,000 | 1:2,000,000 | 1:5,000 |  |


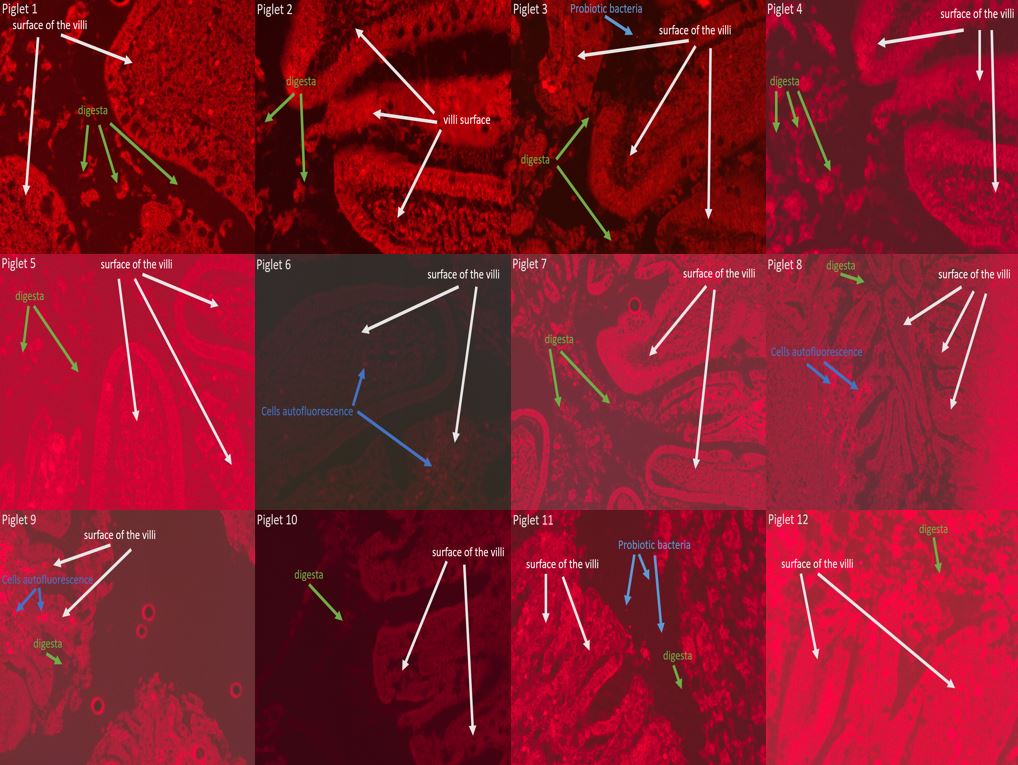


**Fig. S1.** Visualization of the spatial organization of *Bacillus subtilis* and *Bacillus amyloliquefaciens* bacteria in the piglet gut structures sampled from the control piglets (piglets referred to as 1 to 12) that were weaned from pigs that received no probiotic treatment. Samples were analysed by fluorescence in situ hybridization (Histo-FISH) for the presence of probiotic bacteria. All probes were Txrd-labelled (red). White arrows indicate villi surface area, green arrows indicate digesta in the gut, and blue arrows indicate probiotic bacteria or cell autofluorescence.


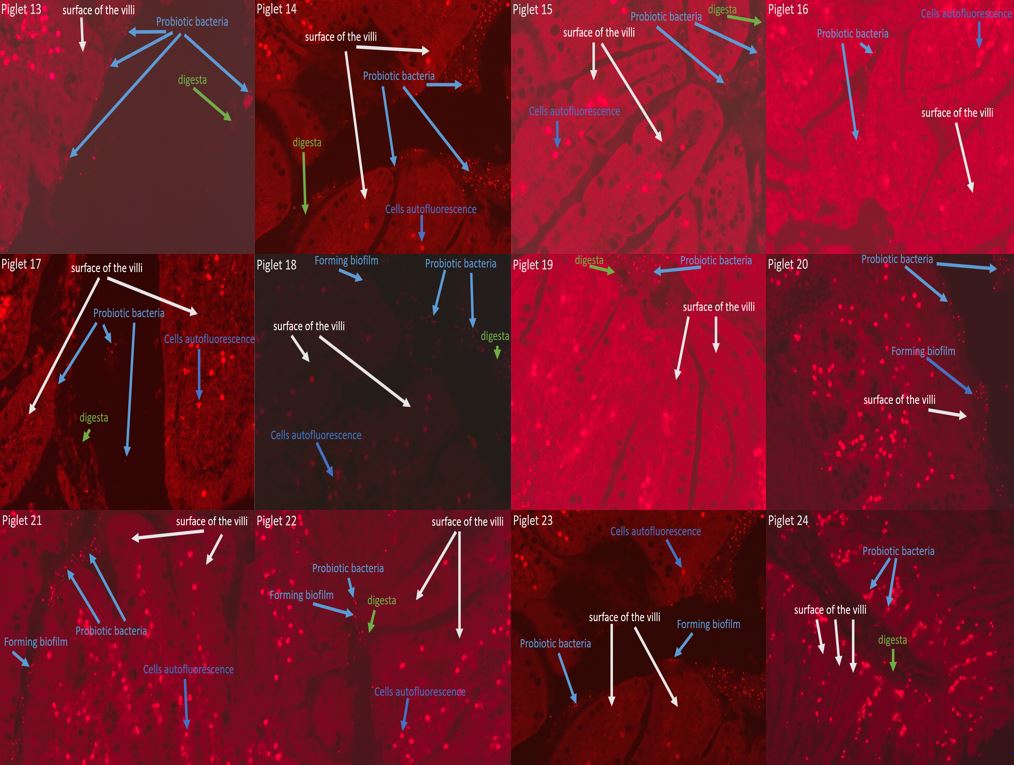


**Fig. S2.** Visualization of the spatial organization of *Bacillus subtilis* and *Bacillus amyloliquefaciens* bacteria in the piglet gut structures sampled from the experimental piglets (piglet referred to as 13 to 24) that were weaned from pigs that received probiotic treatment. Samples were analysed by fluorescence in situ hybridization (Histo-FISH) for the presence of probiotic bacteria. All probes were Txrd-labelled (red). White arrows indicate villi surface area, green arrows indicate digesta in the gut, and blue arrows indicate probiotic bacteria or cell autofluorescence.
